# Management of information within emergencies departments in developing countries: analysis at the National Emergency Department in Benin

**DOI:** 10.11604/pamj.2016.24.263.9370

**Published:** 2016-07-21

**Authors:** Yolaine Glèlè Ahanhanzo, Alphonse Kpozehouen, Ghislain Sopoh, Charles Sossa-Jérôme, Laurent Ouedraogo, Michèle Wilmet-Dramaix

**Affiliations:** 1 Public Health Regional Institute, University of Abomey-Calavi, Benin; 2 Center of research in Epidemiology, Biostatistics and Clinical Research, School of Public Health, Université Libre de Bruxelles, Bruxelles, Belgium

**Keywords:** Emergency Service, hospital, health information, management information systems, Benin

## Abstract

**Introduction:**

The management of health information is a key pillar in both emergencies reception and handling facilities, given the strategic position and the potential of these facilities within hospitals, and in the monitoring of public health and epidemiology. With the technological revolution, computerization made the information systems evolve in emergency departments, especially in developed countries, with improved performance in terms of care quality, productivity and patient satisfaction. This study analyses the situation of Benin in this field, through the case of the Academic Clinic of Emergency Department of the National University Teaching Hospital of Cotonou, the national reference hospital.

**Methods:**

The study is cross-sectional and evaluative. Collection techniques are literature review and structured interviews. The components rated are resources, indicators, data sources, data management and the use-dissemination of the information through a model adapted from Health Metrics Network framework. We used quantitative and qualitative analysis.

**Results:**

The absence of a regulatory framework restricts the operation of the system in all components and accounts for the lack and inadequacy of the dedicated resources.

**Conclusion:**

Dedication of more resources for this system for crucial needs such as computerization requires sensitization and greater awareness of the administrative authorities about the fact that an effective health information management system is of prime importance in this type of facility.

## Introduction

The reception and handling of emergencies is one of the core functions of hospitals, regardless of their level of intervention in the health system and whatever their status. The mission of an emergency department is to receive and provide medical care to anyone in a health emergency condition, without discrimination, 24 hours a day, 7 days a week, especially in case of distress and life-threatening emergency [1]. The challenges which emergency departments have to face daily, in the specific context of rapid action to remedy the life- threatening emergency, are numerous. These include in particular, the quality and promptness of the medical care, the reduction of waiting times, the optimization of the resources available and the satisfaction of the users [2–4]. At the heart of the strategies implemented to address these challenges, stands the management of health information, essential in decision-making for the clinician and the manager [5–7]. The real-time availability of the health information is fundamental in emergency departments: on the one hand for the promptness of individual decision-making by the doctor for the patient’s medical care, and on the other hand, in a more strategic perspective, for the management and improvement of the department performance. With the technologies of information and communication, the management of information was revolutionized in hospitals and emergency departments [8, 9]. So, the availability and installation of certain tools help to facilitate and optimize the medical care provided to the users, but also to monitor and improve the performance of emergency departments through increased productivity, reduced risk of errors and dissemination of the information [5, 10, 11]. While this technological revolution at the service of performance in emergency care units is widely shared by developed and emerging countries that are currently working on other issues, the situation of developing countries is not well documented [12, 13]. The different assessments of the health system in Benin showed that the country is faced with significant challenges, especially, the populations’ access to quality care, which is in connection with major deficiencies in all components of the system, including the management of health information [14]. Benin committed itself, for the period of 2009 to 2018, to the improvement of the quality of care to the populations, including through the development of the hospital sector [14, 15]. In order to propose appropriate strategies, it is necessary to question the management of health information in the emergency departments of Benin hospitals. Accordingly, this study analyses the information management system in the emergency department of the first reference hospital at the national level in Benin.

## Methods

**Scope of the study**: This study was conducted in the Academic Clinic of Emergency Department (ACED) of the National University Teaching Hospital of Cotonou. The National University Teaching Hospital of Cotonou was created in 1962, with a triple mission of permanent reception and medical treatment of the populations, of training and research. It is the largest academic hospital and the main reference facility at the national level. It is a public hospital with a legal personality, and which enjoys financial autonomy under the responsibility of a Board of Directors [16]. It has several departments such as the Academic Clinic and Emergency Department. It is a department designed for the reception and management of emergencies. It features a reception unit, a sorting and orientation unit with 03 boxes of examinations, a shock-treatment unit with 02 beds. It also has an admission capacity of 50 beds for surgical and medical hospitalization. It also has 03 functional operating theaters and an intensive care unit with 06 beds. [17]. That department has a health information collection system, organized around the patient’s records and involving the various service providers of the facility.

**Type of study**: this is a descriptive and evaluative cross-sectional study.

**Study variables**: An adaptation of the conceptual framework of the Health Metrics Network proposed by the World Health Organization for the evaluation of health information systems was used as a reference [18–20]. Therefore, our analysis focused on the following components: the resources: Availability of a leadership and an information policy, Availability and functionality of a physical infrastructure and use of information and communication technologies, availability of human and financial resources; the indicators: Availability of an identification procedure for information needs in the facility, and availability of a list of indicators selected in the facility for the monitoring of activities; the sources and data management: Availability of a system of collection, transmission, processing, and analysis of data, and the quality of the data have been assessed. Data quality assessment was performed through the filling quality of the available tools. Data from the activities of the 1st semester of 2015 were considered on the basis of completeness (most used quality criterion [21]) of four key performance indicators of emergency management units which have been selected: the time before the first consultation, the average duration of the first consultation, the average length of stay and the mortality [5, 22, 23]; the information outputs deriving from the management of data: Availability of a process for their use and dissemination. A summary of the analysis of the Strengths, Weaknesses, Threats and Opportunities was also made.

**Data collection techniques and tools**: This analysis was essentially based on a literature review with counting sheets. This literature review was supplemented by in depth interviews of resource persons with a structured interview guide. The tools used were designed on the basis of the components explored. We used a questionnaire to assess data quality from patients’ records.

**Sampling**: All relevant documents available have been used for literature review. We used the activity reports of the department for the three last years, the patient’s records, memos and different normative documents available. For a data quality assessment, a sample of patients’ records for the 1st semester of 2015 was used. The minimum size n of the sample was calculated by the formula of Schwartz. 2195 cases were registered for the period. For p = 50%, i = 5%, i= 0.05 and = 1.96 Zα, n = 384 records; p is the expected proportion of good quality records, i the accuracy, α the consented risk of error and Zα the standard score of the consented risk. By providing a margin of 10% of unusable records, we estimated the sample size to 420 records. The records were selected by systematic random selection, after calculation of the sampling rate that was 5 or 2195/420. Thus, 438 records were selected. As 25 records were unusuables, finally 413 records were utilized. The targets of the depth interviews were selected by rational choice. It includes the head of department, the head of statistics department, and the representative of the hospital management. The health workers, who were present during the survey and gave their consent, were selected by convenience and interviewed.

**Data analysis**: We used Epi 7 software and Excel for data entry and analysis. We used classic descriptive parameters (percentage with confidence Interval at 95%). For the qualitative part, we proceeded with a thematic content analysis for the results of interviews.

## Results

During the survey, 11 people were interviewed and 413 records were assessed for data quality.

### Resources

The department has no document regulating data collection, either internally or from a higher hierarchical origin. Therefore, the national health information system provides no specific framework for emergency departments within hospitals. The existing infrastructure is not computerized. The collection system is based on form filling of different well-defined materials. An internet connection is available in the department. There is no database management mechanism and there are no computers dedicated to this activity. The financial resources available are integrated into the general operating budget of the department. There’s no budget line dedicated to data collection. Visible inputs which are printed version of different collection materials are provided by the general management of the hospital that ensures regular supply of these materials. The proportion of the budget dedicated to data collection in the department is indefinite. However, the unmet needs are clearly expressed, especially concerning the computerization of data collection. *According to an interviewee, “Without computerization of records, henceforth, we can no longer use the information we collect.”’*Regarding human resources, all the care providers of the department are involved in the data collection through the filling of the different materials provided. They are: 03 intensive care anesthetists, including the head of department, 03 general surgeons, 01 neurosurgeon, 02 general practitioners, 49 nurses, including 03 unit managers. The team of service providers is supported by 20 attendants. There is no staff specialized in the management of information. There is a specialized nurse, unit and statistics department manager. There is a training process for any new service provider upon his arrival in the department, about the filling of various materials. There is no specific motivation system in relation to data collection. Data-collection activities are coordinated by the person in charge of the department statistics. There is no connection with the collection of information in other departments within the hospital and there is no connection with the national health information system, especially with the epidemiological surveillance system. *"There are not enough resources for the department information system because nobody cares"*. According to the person in charge of statistics,*"On my own, I cannot do it anymore in addition to my other responsibilities in the department, I would need some help"*.

### Indicators

The different forms designed provide data relating to the patient’s clinical, paraclinical, therapeutic and progressive data within the department. One can be provided with some standard indicators expected from an emergency department, such as the emergency performance indicators (length of stay), indicators of the impact on the hospital (number of consultations), the indicators relating to ambulatory patients (length of stay), sorting performance indicators (durations of first consultation). Indicators of quality such as the percentages of return are not documented. Moreover, there is no monitoring dashboard for the indicators and there is no normative document on the choice of standard indicators to be collected in the department.

### Source and data management

The data come mainly from the records of the patients. The various tools provided are the admission form, with the information upon reception of the patient, the clinical record, the care sheet, and the therapeutic chart. These different forms are filled by the service providers involved, at their respective levels, along the patient’s circuit in the department until its discharge. All the information gathered through these various materials is compiled in a single file which is forwarded to the head of statistics department, who handles the archiving. The archives are stored in a room intended for that purpose. Access to the archives is limited to the persons authorized by the Head of department and to the staff of the department. There is no regular procedure of processing and analysis of the data collected. Some statistics are published by year end and are mainly related to the indicators of impact on the hospital: number of consultations, number of hospitalizations. There is no actual information circuit formalized with the definition of the different data management levels expected and the actors involved. Regarding data quality, completeness of the data required for the calculation of indicators, is about 75% for mortality, 84% for data needed for the calculation of the length of stay and 55.7% for the data needed for obtaining of time before the first consultation. The data needed to calculate the average duration of the first consultation are comprehensive in 18.6% of records (Table 1).

**Table 1:** Completeness of the data required for the calculation of indicators (n = 413)

Indicators	Data needed	Number of patients records with complete data	Completeness % (CI95%)
**Time before first consultation**	Arrival date and time; First consultation date and time	228	55.2% (50,2-60,0)
**Average duration of the first consultation**	First consultation beginning date and time; First consultation ending date and time	77	18.6% (15,1-22,8)
**Average length of stay**	Arrival date; discharge date	347	84.0% (80,0-87,4)
**Mortality**	Patient status at discharge	309	74.8%(70,3-78,9)

### Dissemination and use of data, Information outputs

The information resulting from the data processing remains in the form of statistics. The process of transformation into factual outputs is still insufficient. The major use of the data is made in response to the regular institutional demand from the hospital administration, in order to elaborate activity reports. Internally, the use of data in the decision-making at the patient’s individual level is made as part of the medical care. All of the patient’s data is then also transmitted to the department where he is transferred, for the continuation of his medical care. Upon request from another service, the patient’s data may also be made available either for the patient’s care or for research or training purposes. Regarding the service itself, the analysis and use of these data in decision-making for the improvement of performances is less obvious. The dissemination is mainly through the department activities reports and research papers on studies conducted within the framework of students’ thesis and dissertation. There is no feedback form. Table 2 summarizes this analysis with a focus on the internal and external environment.

**Table 2:** Analysis of the internal and external environment

	Strengths	Weaknesses	Opportunities	Threats
**Resources**	Availability and training of human resources, Availability of a designated person in charge of statistics	Absence of normative regulatory framework, Lack of budget dedicated to the management of information, Absence of computerization system	Hospital reform at the national level	Lack of awareness of the administrative authorities about the importance of an efficient information system
**Indicators**	Availability of collection tools, Availability of patient records	Absence of dashboard, Lack of a procedure of consensus about the choice of indicators for activities monitoring	Existence of functional consultation frameworks within the department (weekly staff, staff meetings)	Abundance of possible indicators and diversity of profiles of the actors involved
**Data management**	Standardized collection process, Regular archiving, Relative secured storage	Absence of regular data processing and analysis procedure Insufficient quality of data	Availability of qualified personnel (statistician) to equip the department within the hospital	
**Use and dissemination of the information**	Annual issuance of statistics	Inadequate use in managerial decision-making	Status of Training and research centre of the ACED and the hospital	

## Discussion

### Statement of principal findings

*Resources:* the absence of legislative environment is a major deficiency in the functionality of this system, as a result of the predictable negative consequences in terms of planning, coordination and performance. This absence is attributable to a certain negligence of the importance of health information, despite the paradox of a significant evolution of the technologies of information and communication. Regarding availability of both financial and material resources, the analysis of the current situation shows already this lack of material and financial resources that can be explained by the low awareness of the importance of health information by hospital administrative authorities. Regarding human resources, the availability of service providers, users of the system, who also contribute to feed the system, is a positive point. The process of training brand-new staff for the filling of the different forms is also a positive point that has all its importance in a system where the quality of the data collected is an issue for appropriate decision-making. The absence of at least one specialized personnel such as a statistician for example, can prove to be a weakness especially, as this analysis has highlighted in the compilation, in the processing and analysis of data because the data management is here entrusted to a service provider that is also overloaded with other activities. However, such staff may be shared with other services to provide expertise while overseeing the activities carried out by the service providers.

*Indicators:* Improving the performance of an emergency reception and handling facility, requires an objective control allowing different actors to self-assess for the purpose of taking appropriate corrective actions. This analysis at the ACED shows the real basis defect for piloting in the view of improving the department performance.

*Data management:* It’s the formalization of a circuit of information, the different levels of data collection, the various factors involved at each level and the tasks set at each level, all for the optimum quality of data collected. In this study, the lack of formalization of the different stages of data management explains the different difficulties encountered in that department and the poor quality of the data collected.

*Dissemination and use of the information:* The purpose of data collection remains the factual decision-making. The use of the data and their dissemination is still inadequate in the department. Although at the individual patient level, the strategic decision making is more or less effective for planning and performance improvement, it does not make the best possible use of the system outputs.

### Discussion of important differences of results

The strategic position of emergency departments as “public health observatory” is an opportunity to seize, especially in terms of information for the epidemiological surveillance system [24]. The ever common experience in other countries has shown its relevance and effectiveness [25, 26]. Although actual hospital information systems are not yet functional in the country, a formal legal framework must be defined for the collection of information within the emergency reception and handling departments, in order to facilitate the coordination and a more effective use of the information collected. The existence in Benin of a specific legal framework for the epidemiological surveillance system and a relatively well-established national health information system is to be capitalized in this regard [14]. Although the development of computerized health information systems is not easy nor free of problems [27], all the benefits related to the computerization of the emergency departments have been demonstrated, and for many years [5, 8, 28, 29]. In France, in 2013, 86% of emergency departments were computerized [30]. Generally, hospital information systems were experienced even in the developing countries with more or less success [31]. However, major challenges remain, especially as regards the political context, the normative regulatory environment, the technological infrastructure and its implications with other existing systems, the human resources training, etc…. These different components are to be taken into account through a feasibility study in order to consider the implementation of a genuine hospital information system that goes far beyond the simple collection of data as it is currently performed in the department. In the current context of the functionality of a national repository for health information (District Health Information Software-DHIS 2) in the national health information system, the interoperability of databases must for example be considered. Moreover, since the hospital itself does not have a hospital information system (HIS), this parameter must be integrated in the feasibility study to design an integrated and scalable system that can meet the needs of the ACED, while taking into account its external environment. A major challenge remains a greater awareness of the administrative authorities of the Hospital about the importance and sensitivity of health information in general and more specifically of health information in the emergency department. This awareness is the starting point for an adherence of the stakeholders to the evolution of the system through a project of implementation of a Health Information System or of the computerization of the data collection system. Without this adherence, the availability of both financial and material resources can prove to be a bottleneck. However the hospital reform underway for ten years in most countries of sub-Saharan Africa had already identified the health information systems as one of the 3 priority areas, and numerical integration is at the heart of the concerns of the hospitals in the region [32, 33]. Benin, like many countries in the region has already committed to this hospital reform since 2009 [15]. This can be an asset for the implementation of a HIS project in mobilizing the necessary resources. In this regard, a better control of the system costs would undoubtedly facilitate the raising of funds and it is therefore essential to provide tools in order to estimate the costs associated with the functionality of such systems which constitute a significant part of the facilities budget [30]. Stone-Griffith et al. showed in the United States that the use of dashboards improves the performance of emergency departments. [34]. The indicators conventionally used in this type of facilities are numerous, and remain to be adapted according to the capacities to offer care, the context and the objectives set by the department [5, 22, 23, 35]. It is therefore up to each facility to select its priorities and indicators to create its dashboard that becomes at that moment its compass.

A choice of indicators agreed with all stakeholders of the department, including service providers, hospital authorities and care recipients, would be a starting point that will probably evolve with the experimentation. Moreover, this exercise performed in relation with the objectives of the hospital, or even objectives at the national level, will facilitate the integration of these indicators to other hierarchically higher health information systems. Clinically, the treatment of patients could also be enhanced by the identification of clinical indicators selected by the service providers [36]. About, data management, the results of this study are similar to those of the various surveys conducted especially in developing countries and which reveal many problems related to data management. These are mainly organizational problems, data management coordination and data quality problems [14, 18, 19, 37]. With regard to data collection, although the filling of forms is made according to the patient circuit, the role of each player not being clearly defined, the completeness of data poses real problems as revealed by our results. The lack of real-time analysis of the data collected is a significant limit to the improvement of the department performance. As Lindberg shows in the United States, real-time data analysis can contribute to some improvements including reducing the cost per case handled, the level of patient satisfaction [38]. The computerization of the system will both facilitate the use and dissemination of data on more than one ground. On the one hand, the use of tools that have made their worth, e.g. decision trees, can optimize the patient’s medical care through standardization and promptness of the patient’s medical care [36]. On the other hand, the compilation of performance monitoring indicators in dashboards, provides a daily view of the performance of the department [28, 34]. Furthermore the computerization allows a faster and wider sharing and dissemination of data, with the current technological revolution and the emergence of different networks. The reality of the low use of health information generated by data collection systems is similar to that of most health information systems in developing countries, where the weak link remains the insufficient use of data in decision-making [19, 39, 40]. It is therefore necessary to set up within the department, a framework and various procedures for the use and dissemination of information deriving from the activities of the facility, at the service of the improvement of the quality of the medical care given to the patients. The special status of research and training centre of the ACED is also an additional opportunity to remedy this situation, through the implementation of various research projects within the facility.

### Limits of the study and future research

The study is an analysis of a specific department of emergency, therefore it’s not representative of the situation at national level although the ACED is in principle, the most equipped and organized of the emergency departments of the country. So, to provide more information about others emergency department and help strategic decision about health information system enhancement in the emergency department of Benin, it’s a need to conduct further research in the topic.

## Conclusion

This study shows that information management is of prime importance within emergency departments. It reveals that despite availability of a more or less functional overall structure, there is a lack for fundamental resources as legal and official framework, which is basis for all improvements implementation. More joining is need from administrative and political authorities for integration of the nation within this technological dynamics for a higher quality of health services addressed to the population.

### What is known about this topic

- Health Information System are essential for the emergency department;
- The performance of the Health Information System is based on key components as resources, indicators, data management and use of information.

### What this study adds

- The legal framework of the Health Information System in emergency departments in Benin is very weak;
- As a consequence, the resources dedicated are inadequate and the performance are insufficient.
